# CLINICIAN PERCEPTIONS OF UNMET SOCIAL NEEDS AMONG OLDER VETERANS: THE ROLE OF RURALITY

**DOI:** 10.1093/geroni/igae098.0945

**Published:** 2024-12-31

**Authors:** Molly Ream, Laura Polacek, Jennifer Moye, Kelly O’Malley, Lola Baird, Hannah Bashian, Anica Pless Kaiser

**Affiliations:** VA Boston Healthcare System, Boston, Massachusetts, United States; VA Boston Healthcare System, Boston, Massachusetts, United States; VA Boston Healthcare System, Boston, Massachusetts, United States; VA Boston Healthcare System, Boston, Massachusetts, United States; Boston VA Medical Center, Jamaica Plain, Massachusetts, United States; VA Boston Healthcare System, Boston, Massachusetts, United States; VA Boston Medical Center, Jamaica Plain, Massachusetts, United States

## Abstract

Some older Veterans experience unmet social needs due to factors such as poor health, restricted mobility, and living in rural settings. We surveyed clinicians treating older adults (age>60) at Veterans Affairs (VA) hospitals to 1) determine differences in unmet social needs by rurality, 2) explore clinicians’ perceptions of social facilitators and challenges, and 3) assess clinician interest in delivering a group-based intervention: Enhancing Social Functioning for Older Veterans with PTSD (ESVP). We surveyed 84 VA clinicians who self-reported the percentage of their caseload that was rural, which we dichotomized as rural (>=50% rural; n=52) or urban (<50% rural; n=32). Likert-type items assessed the frequency clinicians encountered unmet social needs. Linear regression assessed urban versus rural differences in unmet social needs. We explored descriptive statistics of social facilitators and challenges and interest in delivering ESVP among rural clinicians. Rural clinicians reported greater unmet social needs among Veterans compared to urban clinicians (lack of social support (t(73)=3.10, p=.003); low social engagement (t(73)=2.05, p=.044); loneliness (t(73)=2.61, p=.011)). Yet, among rural clinicians, there were high reports of social facilitators, including close-knit communities (62%), value in helping others (60%), and resiliency (69%). Rural clinicians reported social challenges including fewer engagement options (83%), distance between people (67%), and value of stoicism (60%). There was high interest in delivery of ESVP among rural clinicians (80%). Rural clinicians reported significantly greater unmet social needs among older Veterans compared to urban clinicians. There was high interest in ESVP, which may enhance social functioning among this critical population.

